# CLUSTERnGO: a user-defined modelling platform for two-stage clustering of time-series data

**DOI:** 10.1093/bioinformatics/btv532

**Published:** 2015-09-26

**Authors:** Işık Barış Fidaner, Ayca Cankorur-Cetinkaya, Duygu Dikicioglu, Betul Kirdar, Ali Taylan Cemgil, Stephen G. Oliver

**Affiliations:** ^1^Department of Computer Engineering,; ^2^Department of Chemical Engineering, Bogazici University, Istanbul, Turkey and; ^3^Cambridge Systems Biology Centre and Department of Biochemistry, University of Cambridge, Cambridge, UK

## Abstract

**Motivation:** Simple bioinformatic tools are frequently used to analyse time-series datasets regardless of their ability to deal with transient phenomena, limiting the meaningful information that may be extracted from them. This situation requires the development and exploitation of tailor-made, easy-to-use and flexible tools designed specifically for the analysis of time-series datasets.

**Results:** We present a novel statistical application called CLUSTERnGO, which uses a model-based clustering algorithm that fulfils this need. This algorithm involves two components of operation. Component 1 constructs a Bayesian non-parametric model (Infinite Mixture of Piecewise Linear Sequences) and Component 2, which applies a novel clustering methodology (Two-Stage Clustering). The software can also assign biological meaning to the identified clusters using an appropriate ontology. It applies multiple hypothesis testing to report the significance of these enrichments. The algorithm has a four-phase pipeline. The application can be executed using either command-line tools or a user-friendly Graphical User Interface. The latter has been developed to address the needs of both specialist and non-specialist users. We use three diverse test cases to demonstrate the flexibility of the proposed strategy. In all cases, CLUSTERnGO not only outperformed existing algorithms in assigning unique GO term enrichments to the identified clusters, but also revealed novel insights regarding the biological systems examined, which were not uncovered in the original publications.

**Availability and implementation:** The C++ and QT source codes, the GUI applications for Windows, OS X and Linux operating systems and user manual are freely available for download under the GNU GPL v3 license at http://www.cmpe.boun.edu.tr/content/CnG.

**Contact:** sgo24@cam.ac.uk

**Supplementary information:** Supplementary data are available at *Bioinformatics* online.

## 1 Introduction

High-throughput technologies in the life sciences generate massive amounts of information by allowing the measurement of thousands of entities simultaneously. However, understanding the underlying biological information and drawing meaningful results from such huge data sets is a challenge. Clustering is among the most commonly employed approaches in the analysis of such data and it attempts to identify entities with similar patterns of occurrence while aiming to reveal the functional relationships between those entities.

A number of algorithms have been developed for clustering analyses and their applicability is highly dependent on experimental design, and investigators not infrequently select a less than optimal clustering algorithm with which to analyse their data. Such a choice may result in an inadequate or even misleading, interpretation of the outcome of a given experiment.

Traditional clustering algorithms, such as hierarchical clustering (Eisen *et al.*, 1998), k-means clustering (Tavazoie *et al.*, 1999) and self-organizing maps (Tamayo *et al.*, 1999) are highly applicable heuristic methods (Yeung *et al.*, 2001), which are very suitable for non-dynamic experimental designs. A major drawback to these methods when applied to time-series data is that they take no account of the fact that successive samples in the series are related to one another. Instead, they consider the data from each successive sample as being independent from the data from all of the other samples in that time-series, and thus ignore important information.

Several clustering algorithms have been specifically designed to analyse time-series datasets. Some of these approaches utilize feature-based similarity instead of point-wise similarity (Phang *et al.*, 2003; Sahoo *et al.*, 2007). Although transforming expression profiles into feature vectors prior to clustering was reported to lead to a faster clustering algorithm through noise reduction in the raw data (Kuenzel, 2010), this methodology permits the loss of information during data transformation due to the presence of unexpected patterns and similarities in the data. Ramoni *et al.* (2002) used a Bayesian method, representing gene expression dynamics as autoregressive equations where each expression measurement was assumed to be a linear function of the previous measurements. However, the effectiveness of autoregressive models decreases when the time-series data are non-uniformly sampled (Möller-Levet *et al.*, 2003). Bar-Joseph *et al.* (2003) modelled gene expression profiles using statistical spline estimation as continuous piecewise polynomial functions. This method requires the user to provide the number of desired clusters as input and it is not suitable for short time-series datasets (Liu *et al.*, 2005). Schliep *et al.* (2003) used hidden Markov models (HMM) to account for the dependencies along the time axis. The shortcoming of HMMs is their ineffectiveness for non-uniformly sampled datasets since they disregard the information on how samples are distributed (Möller-Levet *et al.*, 2003).

In the last decade, Bayesian non-parametric models emerged as another model-based option, which allows superior model flexibility (Sammut and Webb, 2010). The most recognized Bayesian non-parametric models are infinite mixture models, which allow a potentially infinite number of mixture components, which can be adapted based on the supplied input. This is achieved through the use of stochastic processes such as the Dirichlet process (DP) or the Pitman-Yor process (PYP) as priors in the probabilistic model. Their mathematical structure is handled by ‘constructions’ like the ‘Chinese restaurant process’ (CRP) or the ‘stick-breaking process’. These representations allow for derivations of iterative inference methods like Gibbs samplers and Markov Chain Monte Carlo (MCMC) methods (Neal, 2000).

Medvedovic and Sivaganesan (2002) developed a gene expression analysis method based on a Bayesian non-parametric model, where a Gaussian infinite mixture model (GIMM) served as the generative model to represent the assumptions regarding the stochastic data generation process implicitly. A ‘complete linkage clustering’ algorithm was employed to determine the final set of clusters. Having noted the difficulty of the problem concerned, the authors suggested the use of ‘average linkage clustering’ algorithm for making the final clustering decision in their subsequent studies (Medvedovic *et al.*, 2004).

Qin enhanced inference on the infinite mixture model by applying collapsed Gibbs sampling, which is a predictive updating technique to integrate out parameters by calculating marginal likelihoods during each iteration (Qin, 2006). The set of samples in a given gene expression profile are assumed to be independently distributed in this Chinese Restaurant Cluster (CRC) algorithm. Joshi *et al.* extended the infinite mixture model approach to allow for the simultaneous co-clustering of genes and experiments (Joshi *et al.*, 2008).

In our approach to time-series gene expression analysis, we have combined the strength and flexibility offered by Bayesian non-parametric methodology by developing and using an infinite mixture model that is tailored to a particular experimental design. Our methodology is similar to other Bayesian non-parametric methods, but our model is specific to the experimental problem. It is implemented in a framework that combines probabilistic inference, clustering, and multiple hypothesis testing.

We present here CLUSTERnGO: a robust clustering methodology for time-series data and, associated with it, a simple, platform-independent user interface to improve its accessibility by experimental biologists, who play a key role in the analysis of such datasets. The methodology assumes a user-defined Bayesian non-parametric model, where each mixture component is modelled as a piecewise linear sequence (PLS) in order to capture the ‘segments’ of time points that comprise the experiment. A two-stage complete linkage clustering procedure was employed to identify the patterns in the data. Unlike its predecessors, this simple and effective approach can address all of the following issues simultaneously: (i) it allows the user to construct their own model, which would integratively take into account both the design of the experiment and the collected data, prior to analysis, (ii) it has a deterministic clustering output, despite its probabilistic approach introduced by two-stage clustering, (iii) it takes into account the differences and the similarities in both the profiles and the magnitudes of expression, (iv) it is suitable for equally or unequally sampled long or short time-series datasets, (v) it does not require an *a priori* knowledge or assumption on the number of clusters that will be identified at the end of the process, (vi) it allows the assignment of the same gene into different clusters, i.e. overlapping clusters, minimizing the loss of biological information hidden in the dataset introduced by two-stage clustering, and (vii) it has a very friendly GUI suitable for both specialist and non-specialist users despite the rigorous computational procedures running in the background.

We test the applicability of our approach on three independent published biological datasets, which are different in size, the level of gene expression under investigation, the temporal experimental design, the presence of replicates, as well as the level of complexity of the model organism and demonstrate that our algorithm brings substantial novel insight into the systems under investigation, which was previously not reported and outperforms its predecessors in doing so.

## 2 Algorithm

The algorithm we propose involves a single process of clustering analysis and consists of four successive phases: configuration (CONF), inference (INF), clustering (CLUS) and evaluation (EVAL) (Supplementary Fig. S1). Inputs and outputs of these operations follow successive steps in a single pipeline. The process, taken as a whole, receives an input dataset of dynamic profiles and assigns the profiles into an optimal number of clusters based on the model determined by the user as well as reporting an output of statistically significant Gene Ontology (GO) terms that characterize those clusters of entities, whenever applicable. In this section, we describe the functioning of each of the four phases in the CnG algorithm pipeline.

### 2.1 Configuration phase (CONF)

The most important feature of datasets on transitions is the dependence of the value of each variable on its value at the preceding time point. Therefore, it is important to account for this information during the identification of clusters of entities displaying similar behaviour over time. Our approach involves building a model based on the experimental input as well as the initial design of the experiment to account for the dependencies between consecutive time points in dealing with transient phenomena. CONF is the phase in our algorithm that configures this model.

**Fig. 1.**
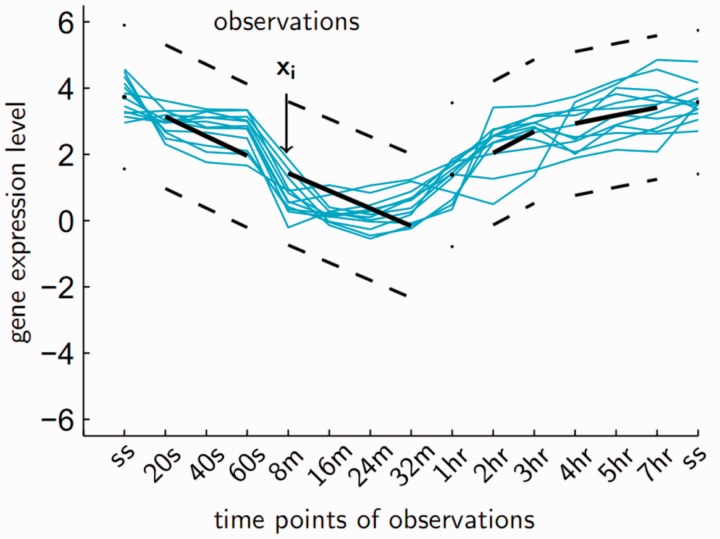
An example piecewise linear sequence model

Our algorithm models the given time-series dataset by an infinite mixture of piecewise linear sequences (IMPLS). IMPLS is a special infinite mixture model whose mixture components are distributed around piecewise linear sequences (PLS). PLS assumes a particular segmentation of time points, where in each segment corresponding to a given time period, the measured level of the clustered entities is assumed to linearly increase, decrease, or constitutively stay constant. PLS model is illustrated in Figure 1.

CONF is the initial phase for configuring the probabilistic model for Bayesian inference. It can be configured manually by specifying a custom segmentation of time points for the PLS model, or it can be configured semi-automatically. In the semi-automatic mode, it takes the time-course profiles of the biological entities in the dataset as its input and, by applying temporal segmentation (TS) to its time points, produces the piecewise linear sequence (PLS) model that will be used in the next phase. TS has a single parameter: the segmentation threshold.

TS determines which time samples show similar behaviour by taking values for each of the time points over the whole dataset, and running a standard average-linkage hierarchical agglomerative clustering procedure based on their pairwise Gaussian distances. By applying a threshold on the resulting dendrogram at a certain value, which we call the segmentation threshold, time samples can be grouped such that they make up a piecewise linear sequence. The threshold is determined by the end-user in order to represent the sub-sequences of time points that are known to have a linear succession in the experimental set-up as the temporal segments in the PLS model.

It is possible to trace how the groupings change as the threshold is varied, thus allowing the user to adjust the time segments until the most biologically meaningful segmentation, based on the experimental design, is obtained. The constructed PLS models are then used to determine the probabilistic model in the inference phase. Although one can also take PLS segmentation as a probabilistic variable to be inferred, in CnG, we choose to keep it as a user-defined model parameter.

Biological experiments are usually designed to seek answers to specific questions and have an *a priori* hypothesis to be tested. This hypothesis is taken into account in the design of an experiment to determine the type and duration of the perturbations as well as the sample collection regime. In CONF, the users can construct their own models that integratively take into account both the design of experiments and the data collected from those experiments. Naturally, the *a priori* expectations arising from the initial design of the experiment may not always meet the actual outcome represented by the data generated. Thus this step may assume the role of an integral checkpoint highlighting important intrinsic characteristics of the data. It may: (i) capture novel behaviour emerging from the data that was initially unexpected when designing the experiment or (ii) highlight inconsistencies or inaccuracies within the data caused either by the experiment itself or its design.

### 2.2 Inference phase (INF)

Following the determination of the PLS model for the given dataset in CONF, INF carries out an operation of Markov chain Monte Carlo (MCMC) probabilistic inference to obtain a pairwise similarity matrix. This output matrix holds the information that will be used in determining the clusters of entities. As input, INF takes the dataset and the PLS model as determined by CONF. As output, it produces the matrix of posterior pairwise probabilities.

To generate this matrix, INF runs an MCMC sampling operation using four parameters: the number of chains, the number of iterations in each chain, the number of iterations to be skipped, and the initial values for hyper-parameters. Following the MCMC run, the pairwise similarity matrix is computed by taking averages over all non-skipped iterations over all chains.

### 2.3 Clustering phase (CLUS)

After obtaining a pairwise similarity matrix by probabilistic inference, we still have to determine the exact clusters of entities and apply hypothesis-testing to detect the significant GO terms associated with those clusters, if applicable. CLUS is the phase that takes this matrix and applies a two-stage clustering operation to obtain clusters (subsets) of genes. This operation has two parameters as input: merge threshold and extension threshold, which are used in its two stages. Two-stage clustering may result in different numbers of overlapping or non-overlapping clusters depending on the given thresholds and the similarity matrix. The threshold parameters determine the stringency of the operation; larger thresholds will result in a larger number of clusters with fewer members, representing finer similarity relations, whereas smaller thresholds will result in a smaller number of clusters that represent coarser similarity relations. The resulting clusters are then received by the evaluation phase for hypothesis testing.

### 2.4 Evaluation phase (EVAL)

Multiple hypothesis testing is applied on the clusters in EVAL. This operation requires GO term assignments for all genes and an alpha parameter (a significance threshold) to use in hypothesis testing. For any given cluster, all GO terms that are directly or indirectly annotated with its member genes are considered as possible hypotheses. Each of these GO terms belongs to one of the three categories: cellular component, molecular function, or biological process. EVAL applies multiple hypothesis testing with Bonferroni or Benjamini-Hochberg correction for multiple testing to the whole set of hypotheses comprised of GO terms from all three categories, and the resulting significant GO terms associated with each cluster are reported in the final output.

## 3 Implementation

### 3.1 CONF: temporal segmentation (TS)

TS is a simple operation where segments of time points that display a correlated behaviour are discerned by applying hierarchical agglomerative clustering to the vectors of values over all entities at each time point in the dataset. The resulting dendrogram is divided by a selected segmentation threshold, and the resulting sub-trees are marked as the time segments of the piecewise linear sequence model that will be used in the next phase. The PLS segmentation can also be set manually by the user (Fig. 1).

### 3.2 INF: MCMC for IMPLS

CLUSTERnGO (CnG) models time-course profiles using an infinite mixture of piecewise linear sequences (IMPLS). To compute the posterior of IMPLS, it uses an MCMC procedure.

#### 3.2.1 The IMPLS model

Suppose that we have *N* entities indexed by *i*∈{1,…,*N*} and their profiles *x_i_*, vectors of size *M*, which are to be modelled as distributed around an unknown number of piecewise linear sequences. Mixture component assignments *z_i_* of these entities are assumed to come from a two-parameter CRP, an iterative construction for a PYP:

(1) $$z_{1:N}|\alpha ,d\;∼\text{CRP}\left(\alpha ,d\right)$$

A PLS model is defined by *L* parameters in the following order: initial value, slope of the first segment, jump to the second segment, slope of the second segment, jump to the third segment, and so on. The prior variances of these three types of parameters are given by *V*_init_, *V*_jump_, *V*_slope_. These variances form the diagonal of the matrix ∑_µ_. For every mixture component *k*∈{1,…, *K*} there is an *L*-vector µ*_k_* that defines a PLS with a Gaussian prior:

(2) $$\mu_{k}|V_{\text{init}},V_{\text{jump}},V_{\text{slope}}∼N{(\mu }_{k}|0,\sum_{\mu })$$

Each cluster also has a precision (inverse variance) parameter λ*_k_* with a Gamma prior:

(3) $$\lambda_{k}\;|\;a,b∼{\Gamma (\lambda }_{k}|a,b)$$

Finally, we have the likelihood, which determines that each time-series is distributed according to a Gaussian with mean *C*µ*_k_* and variance 1/λ*_k_*, where k is the mixture component that this sample belongs to. *C* is a constant matrix that is either manually specified or determined semi-automatically by the CONF procedure. This matrix transforms PLS parameters µ*_k_* into the mixture component mean:

(4) $$x_{i}|\mu ,\lambda ,z_{i}∼\overset{K}{\underset{k=1}{\prod }}N{\text{(}x_{i}|C\mu_{k},\lambda_{k}^{-1}I)}^{z_{ik}}$$

*C* is a matrix of basis vectors and each time-series (here, simply a finite dimensional vector) is modelled by *x* = *C*μ + ε. Mean μ is zero, <μ>= 0. The covariance of *x* is thereby <*xx*′> = *C*<μμ′>*C*′ + *R* = *CC*′ + *R.* The matrix *C* is constructed such that typical *x* are Piecewise Linear Sequences − such sequences will have the conditional covariance *CC*′ + *R.*

#### 3.2.2 MCMC inference

A special Markov Chain Monte Carlo (MCMC) procedure was adopted in the analysis of this model due to the presence of matrix *C*, which transforms the parameter vector.

We run Metropolis-Hastings (MH) steps to sample the mixture component precisions λ*_k_* and use these values to run collapsed Gibbs sampling steps to sample *z_i_* by integrating out the mixture component centres µ*_k_.* Our MCMC algorithm consists of three steps repeatedly applied to converge to the target distribution *p*(*x*, *z*, λ, α, *d*, *a*, *b*).

1. For each *k* = 1…*K*, apply MH steps to re-sample λ*_k_* by *p*(λ*_k_*| *x*_1:_*_N_*, *z*_1:_*_N_*).
2. For each *i* = 1…*N*, apply collapsed Gibbs sampling for *z_i_* by *p*(*z_i_*|*x*_1:_*_N_*, *z*_−_*_i_*, λ_1:_*_K_*) using auxiliary variable method for sampling new λ*_k_.*
3. Apply MH steps to sample the hyper-parameters; α, *d*, *a*, *b* by their, respective, non-informative priors 1/α, 1/*d*, 1 and *b.*

The PLS prior parameters *V*_init_, *V*_jump_, *V*_slope_ are each fixed at a sufficiently large number to assign equal probabilities for different PLS parameter values. The user is allowed to interact with the MCMC on the initial values for the IMPLS hyper-parameters, the number of iterations to be carried out, the number of chains or the skip value. The default values for the number of iterations to be carried out, the number of chains and the skip value for the burn-in period were set as 10 000, 20 and 2500, respectively. The number of iterations and the number of chains are kept at high values to help the MCMC inference to more closely approach its stationary distribution. In practice, this enables the INF phase to yield very similar results in successive runs, even though it is based on a probabilistic algorithm. The default initial settings for the hyper-parameters are as follows; *a* = 2.1, *b* = 0.24, *d* = 0.001 and alpha = 100 although these parameters are readjusted during the iterations.

### 3.3 CLUS: two-stage clustering (TSC)

CLUS is a deterministic phase where decisions are based on simple numerical comparisons on pairwise posterior probabilities. Clusters cannot be determined in the INF phase, because data is finite and there is uncertainty in the infinite mixture posterior. The CLUS phase operates on this posterior to decide on the final clusters. The inference results contained in the pairwise similarity matrix are translated into a set of clusters that indicate groups of related entities through the application of a two-stage operation in the clustering phase. The degree of similarity in clustering is determined by two parameters: the merge threshold and the extension threshold.

Let M be the pairwise similarity matrix where *M_ij_* denotes the similarity between entity i and entity j, namely, the posterior pairwise probabilities between these entities as obtained from MCMC. Given this matrix M and the two threshold parameters, two-stage clustering runs as follows:

1. Prepare an initial set Π of 1-element clusters.
2. Choose the cluster pair (*S_a_*, *S_b_*) where the minimum similarity value between any *i∈S_a_* and *j∈S_b_* is the maximum among cluster pairs.
3. Remove *S_a_* and *S_b_*, and insert their union *S_a_* ∪ *S_b_* = *S_c_* into the set of clusters; Π.
4. Continue from step 2 until the obtained similarity between *i ∈ S_a_* and *j∈S_b_* is smaller than the merge threshold.
5. Choose the cluster-entity pair (*S_a_*, *j*) where the minimum similarity value between any *i ∈ S_a_* and *j* is the maximum among all pairs.
6. Remove *S_a_* and insert its incremented set *S* = *S_a_* ∪ {*j*} into the set of clusters; Π.
7. Continue from step 5 until the obtained similarity between *i∈S_a_* and *j* is smaller than the extension threshold.

Among these steps, 2, 3 and 4 designate the first stage where small clusters are merged into larger clusters, and 5, 6 and 7 designate the second stage where clusters are further extended by inserting elements. Intuitively, the merge threshold determines the size of cluster cores, whereas the extension threshold determines the extent of overlap among cluster peripheries. Lowering the extension threshold in stage 2 can result in wide cluster peripheries that overlap for many genes. Lowering the merge threshold in stage 1 will yield few large cluster cores, thereby effectively constraining the possibilities of overlaps in stage 2. Using this methodology, there is no need for any *a priori* knowledge or assumption concerning the number of clusters that will be identified at the end of the process. The default settings for the merge and the extension threshold parameters were both 0.5, although they can be individually set by the user to any value between 0 and 1.

### 3.4 EVAL: multiple hypothesis testing

The identified clusters of genes are significantly associated with a biological ontology through the application of multiple hypothesis testing in EVAL. Gene Ontology (GO), where each gene is annotated by a list of terms from three domains: cellular component, molecular function, and biological process was adopted as the biological ontology in this analysis (Ashburner *et al.*, 2000). To determine if a given cluster is annotated by a given GO term at a frequency greater than by chance, the p-value is computed using the hypergeometric distribution:

(5) $$P=1-\overset{k-1}{\underset{i=0}{\sum }}\frac{\left(\begin{array}{c} M\\ i \end{array}\right)\left(\begin{array}{c} N-M\\ n-i \end{array}\right)}{\left(\begin{array}{c} N\\ n \end{array}\right)}$$

Here, *N* is the total number of unique genes, *M* is the number of genes annotated by the term, *n* is size of the cluster, and *k* is the number of annotated genes in the cluster. Bonferroni correction was used as a conservative action to control the family-wise error rate. Although the Bonferroni correction is set as default, the Benjamini-Hochberg procedure is also provided as a more relaxed option to control the false discovery rate (FDR) at level alpha. The assigned GO term is identified as significant if the *P*-value is less than the significance threshold, whose default was set as α = 0.01.

## 4 Validation

### 4.1 Datasets

We selected three datasets, which were previously analysed using traditional clustering algorithms. Two datasets comprised non-replicate time series gene expression profiles with unequal sampling points. The first dataset (GLU) was generated in a study investigating the response of *Saccharomyces cerevisiae* to an impulse-like perturbation to remove glucose limitation from the culture environment (Dikicioglu *et al.*, 2011) and the second study (SPO) investigated how the transcriptional response varied over time shifting from spore formation (facilitated by the starvation of *S. cerevisiae*) to the germination of those spores induced by their transfer into rich medium (Geijer *et al.*, 2012). Data were available at *t* = 0, 20, 40, 60 s, 8, 16, 24, 32 min, 1, 2, 3, 4, 5, 7 and 80 h in GLU and at *t* = 0, 4, 8, 16, 32, 48, 64, 96 and 128 min in SPO. Both studies reported a subset of genes with a differential transcriptional response (372 transcripts and 1151 transcripts for GLU and SPO, respectively) (File S1). The third dataset comprised of the circadian oscillations of the proteome of *Mus musculus* liver cells (MUS) provided as three independent biological replicate subsets (File S1) (Robles *et al.*, 2014) and data were available for 3089 proteins at equally sampled time points of *t* = 0, 3, 6, 9, 12, 15, 18, 21, 24, 27, 30, 33, 36, 39, 42 and 45 h.

### 4.2 Effect of parameter selection

Our clustering method (CnG) is simple enough to be used by researchers analysing datasets created by dynamic sampling regardless of their experience in high-throughput data analysis, yet it is sufficiently flexible to allow more experienced data analysts to explore their options in detail. The Bayesian non-parametric methodology permits most of the model parameters to be determined automatically or integrated out analytically. User-defined parameters are introduced only as needed and are intended to be kept at minimum in order to avoid an unnecessary increase in the complexity of analysis. The effect of varying these parameters on the outcome of the analysis are demonstrated using the three datasets detailed in the previous sub-section.

**Fig. 2.**
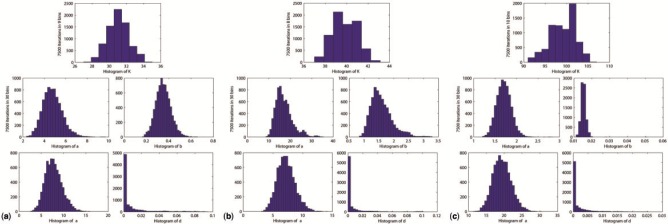
Parameter distribution and convergence. Histograms of *K,* α*, d, a, b* through the iterations for GLU (**a**), SPO (**b**) and MUS (**c**)

A segmentation threshold has to be defined by the user in the CONF phase to mark the sub-trees in the resulting dendrogram as the time segments in which the values display a similar trend in behaviour. This selection depends solely on the nature of the data, the design of the experiment, and the biological question that was sought after. The decision, therefore, has to be taking full advantage of the methodology to extract the most from the dataset. As the user varies the threshold, the segments formed at that threshold are visualized at the same time. This allows the user to adjust the threshold such that the biological system under investigation may be represented as realistically as possible. In the present analyses, the segmentation threshold was selected as 9 for GLU, 19 for SPO and 15.6 for MUS leading to the following segmentation profiles of (1)-(2, 3, 4)-(5, 6, 7, 8) (9)-(10, 11)-(12, 13, 14)-(15), (1, 2, 3)-(4)-(5, 6)-(7, 8, 9) and (1, 2, 3, 4, 5, 6, 7, 8)-(9)-(10 11)-(12)-(13 14 15 16), respectively. The numbers here represent the order of the time points under investigation and the brackets define the clusters that contain the indicated time-points. The similarity matrices obtained for GLU, SPO and MUS in the INF phase, which would then be used in the identification of the clusters in the next phase, had 43%, 44% and 39% of the elements with non-zero values, respectively. The convergence behaviour of our probabilistic inference method employed in the INF phase was explained using the three datasets. We investigated how the number of mixture components and the hyper-parameters; α, d, a and b varied during the MCMC run. The distribution of these parameters is given in Figures 2–c for GLU, SPO, and MUS, respectively. These histograms were computed over 7500 iterations, omitting the first 2500 burn-in iterations. The number of mixture components *K*, and all the hyper-parameters; α, *d*, *a*, *b* are observed to converge toward their target distribution *p*(*x*, *z*, λ, α, *d*, *a*, *b*) and oscillate around their respective marginal posteriors.

The hyper-parameters (*a*, *b*) of the mixture components precisions (λ*_k_*) converge to their respective distributions around 4.5 and 0.35 for GLU, 15 and 1.5 for SPO and 1.7 and 0.015 for MUS. The different distributions for the hyper-parameters *a*, *b* suggest that the mixture components for the datasets are inferred to have different distributions for their precision parameters λ*_k_.* Namely, in the first and the second datasets, precisions λ*_k_* are likely to be distributed around the values 10 and 9 that carry the highest probability; whereas in the third dataset, they are likely to be distributed around the value 47 that carries the highest probability.

The other two hyper-parameters (α, *d*) that determine the non-parametric prior's tendency to create more mixture components, oscillate around (8, 0) and (7, 0) for GLU and SPO and around (19, 0) for MUS. The method infers similar hyper-parameters; α, *d* for these different datasets, thus they are inferred to have similar concentrations in their generative process of partitioning modelled by CRP. The number of mixture components *K* oscillates around 31 for GLU, 37 for SPO and 101 for MUS, implying the presence of more clusters as the size of the dataset increased.

To finalise the INF phase, the information sampled in Bayesian inference is summarised in a pairwise similarity matrix to be passed on to the CLUS phase. The similarity matrices obtained for GLU, SPO and MUS had 43%, 44% and 39% of the elements with non-zero values, respectively.

The CLUS phase in the algorithm hosts the next set of user-defined parameters; the merge threshold (*m*) and the extension threshold (*e*). TSC is a simple yet powerful procedure that enables a threshold-based exploration of possible clusters of entities suggested by the similarity matrix obtained from model-based inference, without making any additional linearity assumptions. The merge threshold determines the maximum number of clusters that can be identified, whereas the extension threshold determines the maximum extent of these clusters being identified. The final number of unique clusters depends on both of these thresholds.

**Fig. 3.**
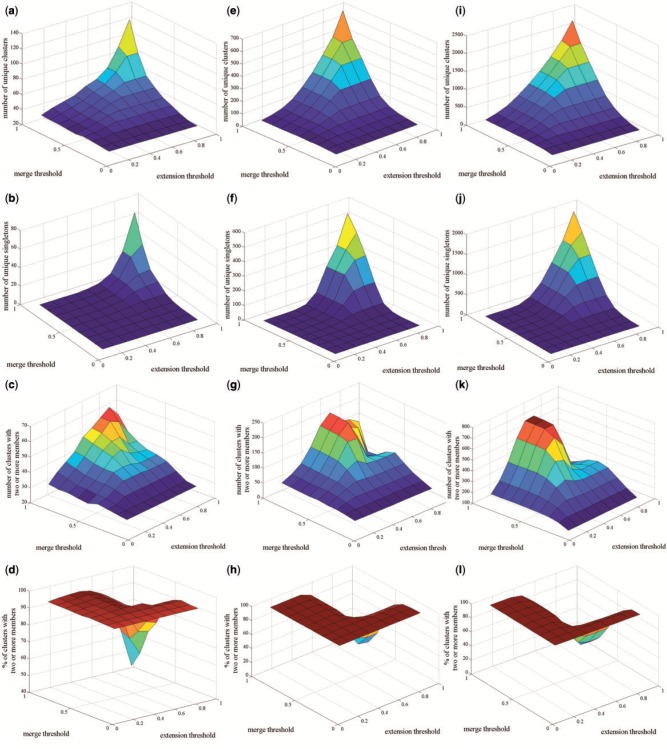
Variation in the number of clusters. The number of clusters (a, e, i), the number of singleton clusters (b, f, j), the number of clusters with two or more members (c, g, k), and the percentage of clusters with two or more members among the total number of clusters (d, h, l), in GLU, SPO and MUS, respectively, as a function of *m* and *e* are displayed. Both the total number of unique clusters and singletons increases as *m* and *e* get higher, whereas the percentage of clusters with two or more members among the total number of clusters begins to drop considerably at values higher than 0.6 for both m and e

We investigated how these parameters affect the clustering structure by varying their values in increments of 0.1 between 0.1 and 0.9 for both the merge and the extension thresholds. We carried out these analyses on the GLU, SPO and MUS datasets (File S2, S3 and S4, respectively). Setting *m* to a low value allowed the clustering process to be less stringent, resulting in a few large clusters; whereas higher thresholds were associated with a more stringent clustering strategy, increasing the maximum number of clusters that can be identified by the algorithm. The size of the dataset under investigation was an important criterion in determining the total number of clusters. The number of unique clusters increased as the dataset got bigger. Furthermore, the size of the largest cluster was observed to be smaller at high e (Table 1, Fig. 3, Supplementary Fig. S2).

**Table 1.** Summary of the clustering analysis of the datasets given at the marginal parameter settings for *m* and *e*

	Merge threshold	0.1	0.1	0.9	0.9
	Extension threshold	0.1	0.9	0.1	0.9
**GLU (372 transcripts)**	Total number of unique clusters	30	30	34	126
	Size of the largest cluster	34	32	34	21
	Number of singletons	2	2	2	70
	Number of clusters enriched with 1 ^+ ^GO term*	13	10	18	12
	% of clusters enriched with 1 ^+ ^GO term**	46	36	56	21
**SPO (1151 transcripts)**	Total number of unique clusters	39	39	52	694
	Size of the largest cluster	127	103	128	30
	Number of singletons	0	0	0	545
	Number of clusters enriched with 1 ^+ ^GO term*	14	10	16	33
	% of clusters enriched with 1 ^+ ^GO term**	36	26	31	22
**MUS (3089 proteins)**	Total number of unique clusters	117	117	184	2249
	Size of the largest cluster	142	121	142	35
	Number of singletons	0	0	0	1906
	Number of clusters enriched with 1 ^+ ^GO term*	2	3	2	18
	% of clusters enriched with 1 ^+ ^GO term**	2	3	1	5

(*) indicates the number of clusters with two or more members, which are significantly enriched with at least one Biological Process GO Term (for GLU and SPO) and Molecular Function GO Term (for MUS) (*P*-value < 0.01). (**) represents the relative percentage of clusters with two or more members.

The merge and the extension thresholds have specific tasks in the algorithm: the former determines the number of clusters, while the latter determines the number of single-member clusters (which we will refer to as singletons). The two parameters interact in a complex manner to determine the number of clusters with a single member within the same dataset. It should be noted that, in general, a high number of singleton clusters may be an undesirable feature of any clustering application and should be avoided whenever possible.

A limited number of highly populated clusters are obtained with low values of *m* and *e*, which would be useful for highlighting the global responses of the biological system under study. However, it is also important to adjust m and e to higher values to obtain many small clusters. These may help in the identification of subsets of biological entities with very similar profiles to a given perturbation, which might indicate subtly different biological responses.

We found the number of singletons to increase with increasing m and e (File S5). This increase was observed to become steeper, especially in the range of 0.4–0.6 for *m* (Fig. 3b, f, j and d, h, l). Therefore, we adopted 0.5, the median of this range, as the default parameter setting for m in CnG. This value allows a sufficiently high number of unique clusters to be identified without allowing a high fraction of them to be populated by only one entity.

The unique clusters with two or more members are of particular interest since such clusters are suitable for annotation with biological ontologies. We therefore investigated how the number of such clusters varied with the extension threshold. The highest number of clusters with at least 2 members was obtained in the range of e values of 0.3–0.6. More of these clusters would be obtained towards the lower end of this range as the datasets got bigger (Fig. 3c, g and k). We adopted a default value of 0.5 for the extension threshold, based on the results we have obtained for the three datasets under investigation. We suggest using high values for both m and e without making compromises to have an elevated fractional representation of singleton clusters and the case studies indicate the suggested default of 0.5–0.5 as a reasonably safe choice. The number of clusters obtained with these settings maintains the optimal balance between having a manageable number of clusters without making substantial compromises on the extent of functional or biological annotation that could be acquired from the clustered entities in EVAL, whenever applicable. However, we strongly encourage users to explore their options with these two thresholds tailoring their analysis to the intrinsic nature of their experiments.

In order to further investigate the dependency between the individual cluster sizes and m and e, we have explored the entire distribution of cluster sizes as a function of these two parameters, at critical combinations of m and e settings; 0.1, 0.5 and 0.9. We also focused on the range around the default parameter settings and conducted an analysis at combinations of m and e, at 0.4, 0.5 and 0.6 (Supplementary File S6, Figs S3 and S4). Our analysis indicated that the large clusters dissolved as the merge and the extension thresholds are increased, giving way to clusters with smaller size and this effect was most prominently observed in the singletons. We also observed that keeping m and e within the suggested range of 0.4–0.6 but selecting other settings than *m* = *e* = 0.5 did not yield substantial differences in the distribution of the cluster size or any compromise regarding an overshoot in the number of singletons. Our analysis indicated that these two parameters affect not only the number of singletons but the entire distribution of the cluster sizes.

We have also investigated the separation of the clusters, exploring the average inter- and intra-cluster distances at m and e settings exploring the possible range of combinations of values. We have observed that the inter-cluster distance remained much higher than the intra-cluster distance for all datasets, at any selected *m* and *e* setting, indicating the separation between the clusters was sufficiently larger than that of the average intra-cluster variance at any selected threshold (Supplementary File S7, Fig. S5). The separation between the average inter- and intra-cluster distances was especially large for GLU and SPO, which comprised only genes that display a significant change in their expression profiles over the transition period. On the other hand, the distance between inter- and intra-cluster variances was observed to be shorter in MUS, where the significance of protein expression levels was not taken into consideration.

EVAL allows the user to employ either Bonferroni correction to control the family-wise error rate or Benjamini-Hochberg procedure to control the false discovery rate (FDR) at a given confidence level in multiple hypothesis testing. Bonferroni correction was observed to yield a stricter evaluation with fewer annotations, which could be attributed to the assigned clusters regardless of the size of the dataset (File S5). The filtered GO annotation files for *S. cerevisiae* (gaf version 2.0—05/04/2014) and for *M. musculus* (gaf version 2.0—09/07/2014) as well as the ontology (OBO v1.2—09/04/2014) files used in the analysis were obtained from the Gene Ontology Consortium webpage (http://geneontology.org/page/download-ontology).

## 5 Discussion

### 5.1 Evaluation of the performance of CnG among model-based clustering algorithms

The performance of CnG was evaluated by comparing the extent of biological insight gained employing this methodology to that gained by two predecessor model-based algorithms, CRC and GIMM. The default settings for CRC and CnG were used in this analysis. GIMM has a user-defined parameter setting, with no initial default value provided, and we adopted the median value for this analysis.

Initially, we carried out an internal evaluation of the clustering results to assess the quality of the set of clusters obtained from CnG in comparison to CRC and GIMM. We determined the intra-cluster tightness and inter-cluster separability based on the Davies-Boudlin index (DBI) (Davies and Bouldin, 1979), where a lower index value indicates better clustering. We computed this index for the complete range of m and e available in CnG as well as employing a range of values for the default setting of CRC and the user-defined setting of GIMM. Our results indicated that, for all test cases, the DBI of CRC algorithms varied in a very small range and the index value at its default setting was equal to that of the lowest value attainable (Supplementary Table S1). CnG and GIMM both provided a wide range of DBI values across a range of parameter settings, with the DBI of CnG remaining lower than that of GIMM even at the setting that would yield the maximum value for the index, indicating higher intra-cluster similarity (distance) and lower inter-cluster similarity (dispersion). Internal evaluation schemes, although providing a validation on how well the clustering has performed, do not necessarily imply the best information retrieval (Manning *et al.*, 2008). Therefore, we next analysed the extent of biological insight gained from the algorithm.

The cluster enrichments for biological process GO terms were used in the evaluation of GLU and SPO datasets in compliance with their respective publications, whereas the cluster enrichments for molecular function GO terms were evaluated for the MUS dataset, as reported. The fraction of overlap in the assignment of unique GO terms to clusters by these algorithms was inspected (Table 2). The results of this comparative analysis indicated that CnG outperformed its predecessors in the extent of the additional biological information that could be attributed to the dataset under investigation. We then proceeded to investigate whether or not the unique GO terms identified only by CnG were only the child terms of a parent that would already be identified by the other clustering algorithm used in the comparison. The investigation of the GO terms by REVIGO (Supek *et al.*, 2011) revealed that the pool of identified GO terms included a mixture of both newly identified terms, thus leading to novel biological information extracted from the data, as well as child terms, helping to reveal more specific information from the dataset under investigation (File S8). A further investigation of the expression patterns observed in the clusters that were attributed with similar functionalities by the three algorithms as well as those for which only CnG could assign functionality indicated that highly populated clusters, at times, fail to capture small clusters with specific functionality attributions and may prove problematic in capturing differences in expression profiles (Supplementary Figs S6 and S7).

**Table 2.** GO Term coverage performance of CnG in comparison to preceding clustering algorithms

	GLU	SPO	MUS
No. of terms identified by CRC in total	43	92	1
No. of terms identified by CnG in total	85	135	10
% of terms CnG identifies in CRC results	91%	82%	100%
% of terms CRC identifies in CnG results	46%	56%	10%
No. of terms identified by CRC only	4	17	0
No. of terms identified by CnG only	46	60	9
No. of terms identified by CnG and CRC	39	75	1
No. of terms identified by GIMM in total	79	64	7
No. of terms identified by CnG in total	85	135	10
% of terms CnG identifies in GIMM results	78%	80%	71%
% of terms GIMM identifies in CnG results	73%	38%	50%
No. of terms identified by GIMM only	17	13	2
No. of terms identified by CnG only	23	84	5
No. of terms identified by GIMM and CnG	62	51	5

### 5.2 CnG clustering platform to get deeper biological insight from the data

Having established that CnG extends the biological knowledge on a system considerably, we then proceeded to investigate if this new information could be used in extending our understanding of the systems that were under investigation by revealing novel insights. We observe that CnG identified more specific child GO terms associated with the clusters and brought novel biological insight into the analysis of the experimental system under investigation in all of the three datasets.

CnG identified a group of genes whose expression was up-regulated in response to an impulse-like addition of glucose in the GLU dataset and associated that cluster significantly with tRNA aminoacylation for protein translation process of the tRNA metabolic process parent GO term. This biological process was not captured in the clustering analysis followed by ontology enrichment analysis in the respective publication. However, the publication reported another tRNA metabolic process, tRNA modification, to be captured through integrative analysis of the transcriptome data with transcriptional regulatory information.

A similar observation was made in the analysis of the SPO dataset. A cluster of genes, which were up-regulated upon the induction of germination by transferring the cells into rich, glucose-containing medium was identified and the cluster was significantly enriched with the glucose transport GO process term. Concordantly, a cluster comprised of genes that were significantly down-regulated was enriched with the gluconeogenesis GO process term. This phenomenon of shifting towards glucose metabolism was identified via the analysis of transcription factors through an integrative analysis of the transcriptome data with gene regulatory information.

These findings indicated that the fine-tuning introduced by constructing a model for the transient behaviour of the dataset allowed clustering to capture subtle features embedded in the data, which could otherwise only emerge through the use of elaborate integrative methods. A total of 147 unique GO terms were significantly associated with the clusters identified by CnG analysis of the MUS dataset, for which 27 metabolic and cellular processes were attributed in its respective publication. In order to be able to evaluate how CnG performs, we focused on the 186 cyclic proteins identified in the publication, for which clustering results were available.

A separate clustering analysis of these cyclic proteins revealed that more specific GO component localizations could be attributed to the proteins that vary according to the circadian rhythm of the organism. Liver proteins that were significantly associated with secretory granules, extracellular vesicular exosomes, blood micro-particles, and platelet alpha granules could be identified as more specific GO component terms in addition to the reported extracellular space and fibrinogen complex. Furthermore, the cluster of daytime-enriched proteins was significantly associated with a novel myelin sheath GO component term. A connection between liver-associated problems (fatty liver dystrophy) and impaired nerve function has long been known (Klingenspor *et al.*, 1999). The present findings indicated that there might be an additional factor introduced into this interconnected mechanism through the relevant proteins’ response to the circadian clock.

Furthermore, GO process and function terms could be attributed to the clusters populated with these cyclic proteins. The processes and the functions that were identified by CnG analysis were inclusive of the KEGG pathways and Uniprot Keywords discussed in the publication as well as pointing out other processes and functions, which might display cyclic responses. The cluster of daytime-enriched proteins was significantly associated with the regulation of the ERK1 and ERK2 cascade process GO term. A previous study reported that the activity of Ras/ERK signalling exhibited circadian rhythms in the mouse liver clock (Tsuchiya *et al.*, 2013).

The clustering of the whole proteome provided as the MUS dataset revealed a cluster of 29 tightly bound proteins and two proteins in this cluster are involved in heme-copper terminal oxidase activity. Although cytochrome c oxidase subunit 6B1 was identified among the subset of proteins that were responsive to the circadian clock, cytochrome c oxidase subunit 7A2 from the same cluster, with a very similar transient expression profile, was not. The CnG analysis suggested the inclusion of this protein among the subset of circadian rhythmic proteins of the mouse liver cell as indicated by the analysis.

These findings indicated that CnG analysis contributed to the better understanding of the biology of the system under investigation by providing novel and detailed insights regarding the dataset.

## Supplementary Material

Supplementary Data
