# Supplementary material for: CLUSTERnGO: a user-defined modelling platform for two-stage clustering of time-series data
Source: Bioinformatics. 2015 Sep 26;32(3):388–97. doi: 10.1093/bioinformatics/btv532 (PMC4734040; doi:10.1093/bioinformatics/btv532)
Supplement: Supplementary Data [file supp_32_3_388__index.html]

CLUSTERnGO: A user-defined modelling platform for two-stage clustering of time-series data — CLUSTERnGO: a user-defined modelling platform for two-stage clustering of time-series data — CLUSTERnGO: a user-defined modelling platform for two-stage clustering of time-series data — Supplementary Data 

# CLUSTERnGO: a user-defined modelling platform for two-stage clustering of time-series data

## Supplementary Data

files

- Supplementary Data - zip file
