# Supplementary material for: CLUSTERnGO: a user-defined modelling platform for two-stage clustering of time-series data
Source: Bioinformatics. 2015 Sep 26;32(3):388–97. doi: 10.1093/bioinformatics/btv532 (PMC4734040; doi:10.1093/bioinformatics/btv532)
Supplement: Supplementary Data [file supp_btv532_suppl_data.zip › BIOINF-2015-0311_SupplementaryFile.docx]

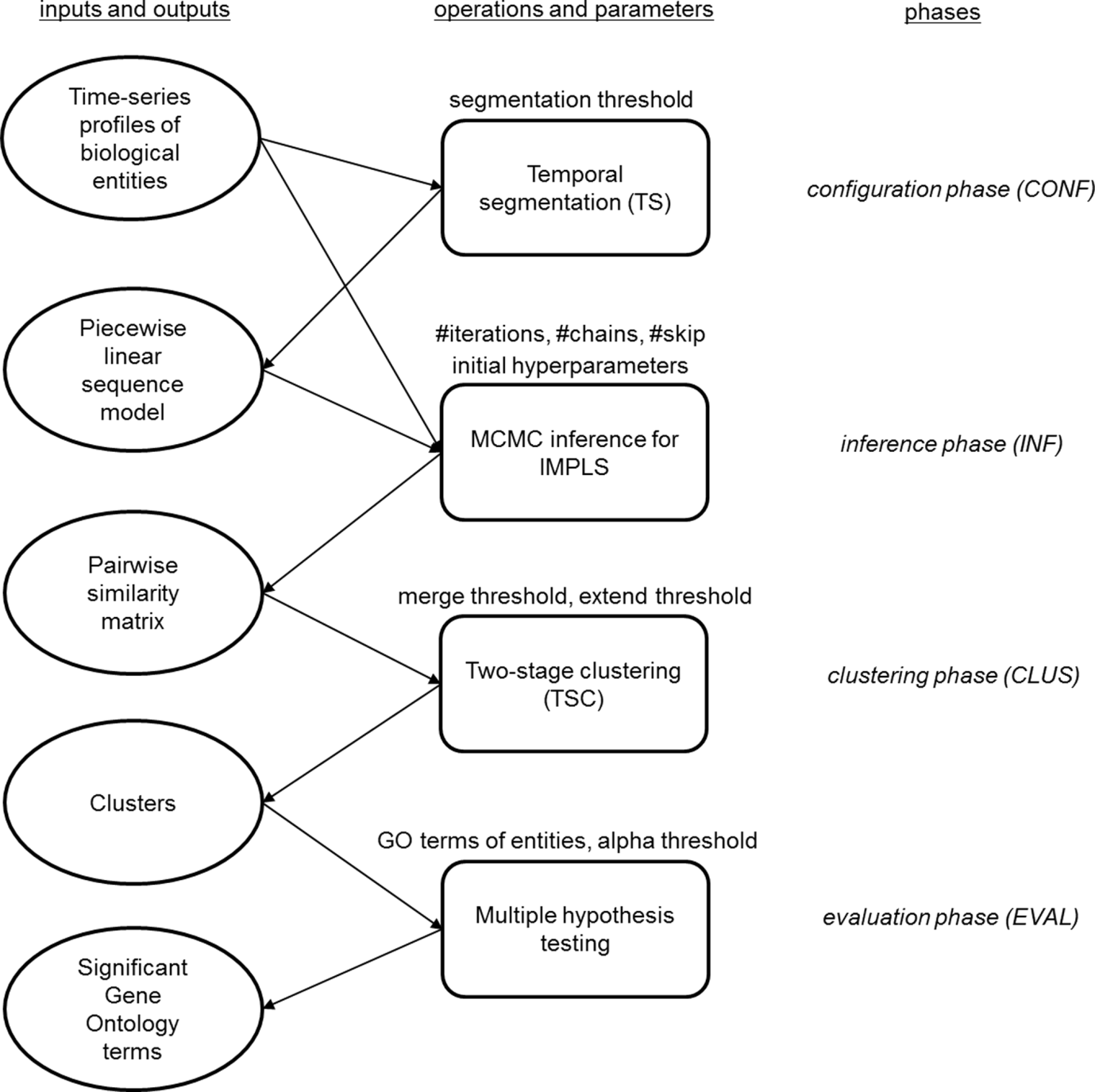


**Figure S1** Structural design of the algorithm. The CAG process consists of four phases: configuration, inference, clustering, and evaluation. Operations in each phase are shown in rectangular boxes, with any parameters (if applicable) written above them. Inputs and outputs of these operations are indicated in oval boxes.


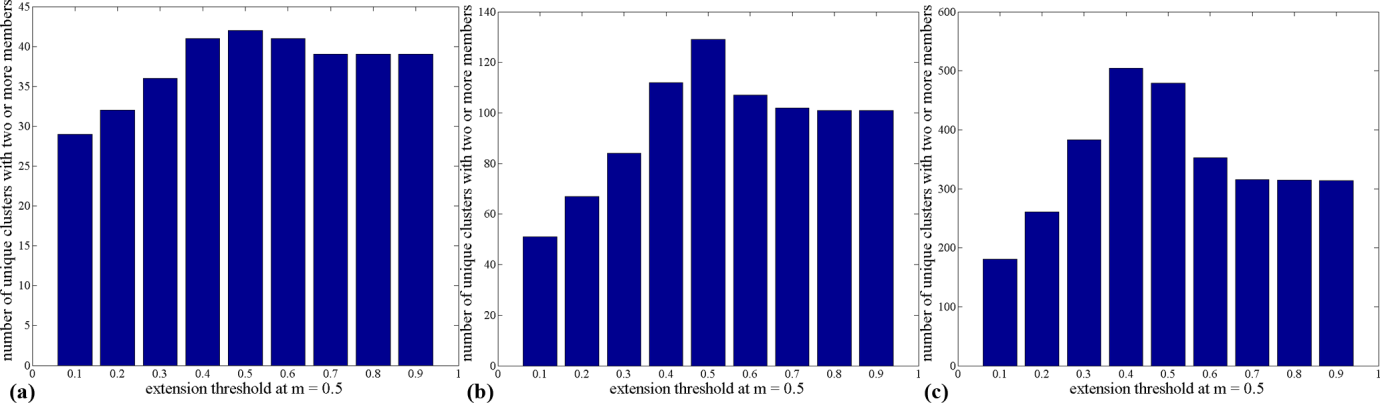


**Figure S2** The variation in the number of clusters (a, b, c) with respect to the extension threshold for a fixed value of merge threshold in GLU, SPO, and MUS, respectively. The number of clusters with two or more members was observed to peak in the extension threshold range of 0.3-0.6 for a given merge threshold, with m = 0.5 employed here for demonstration. This figure is linked to Figure 2 in the main text.

**
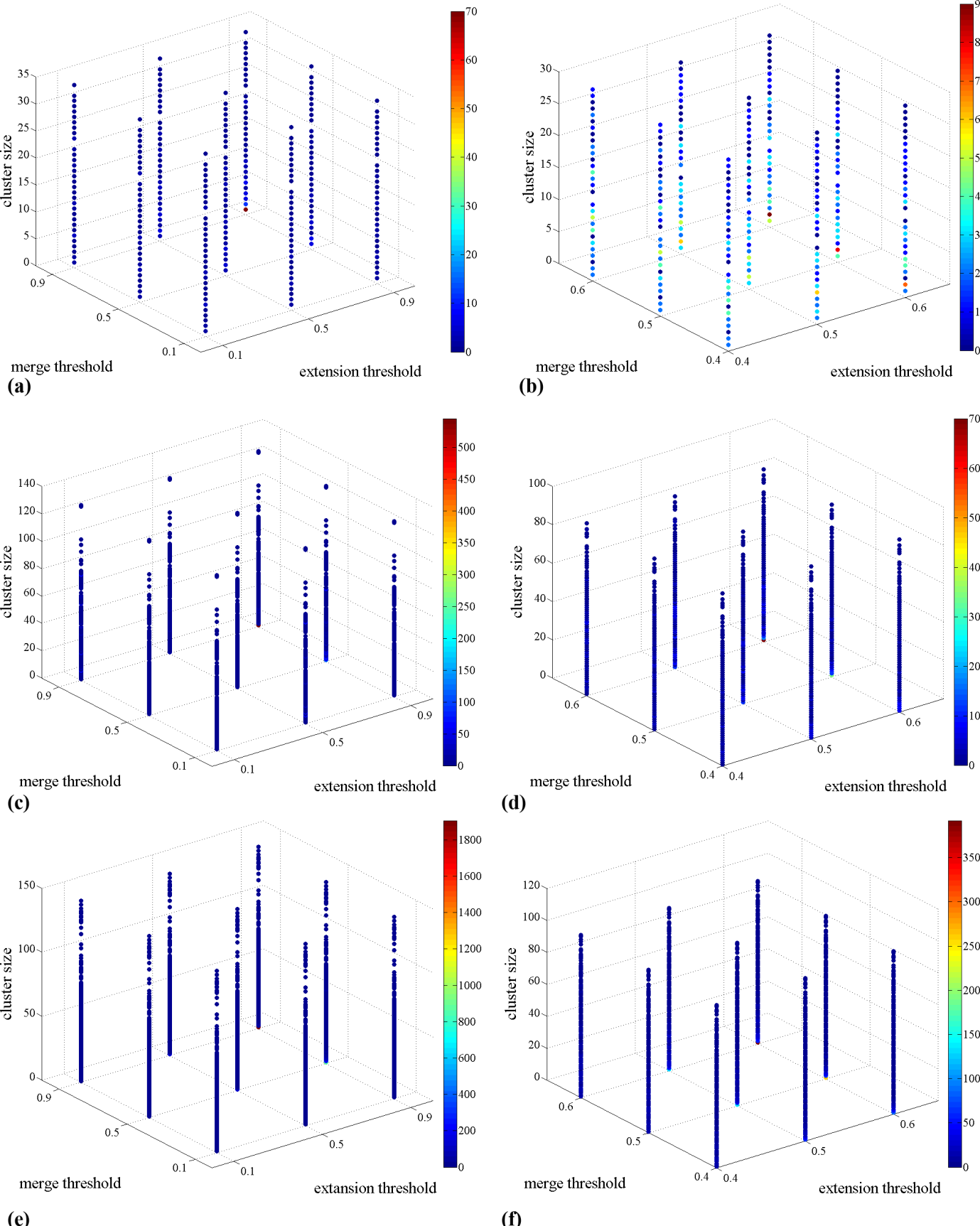
Figure S3** Distribution of cluster size as a function of the merge and extension thresholds for selected critical settings. The variation in the number of clusters of given size are colour-coded. The critical range of threshold pairs were investigated at the extreme values of 0.1 and 0.9 within the context of the default setting in CnG; 0.5 (a, c, e) as well as the thresholds within the suggested range or 0.4 – 0.6 (b, d, f). The results for GLU, SPO and MUS datasets are displayed in (a and b), (c and d) and (e and f), respectively.


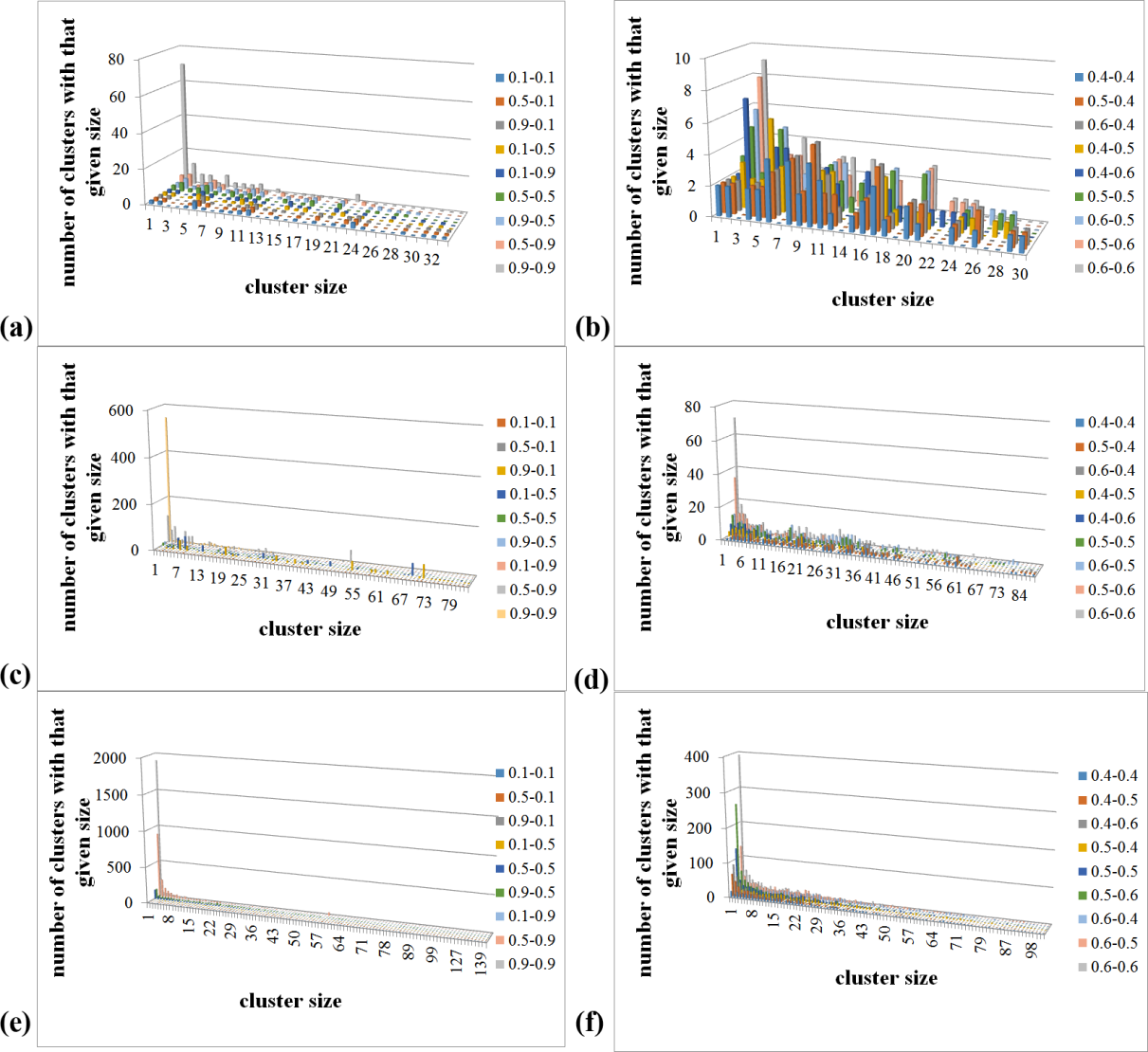


**Figure S4** Distribution of cluster size for critical merge and extension threshold pairs. The variation in the number of clusters of given size are indicated for a critical range of threshold pairs investigating the extreme thresholds of 0.1 and 0.9 within the context of the default setting in CnG; 0.5 (a, c, e) as well as the thresholds within the suggested range or 0.4 – 0.6 (b, d, f). The results for GLU, SPO and MUS datasets are displayed in (a and b), (c and d) and (e and f), respectively.


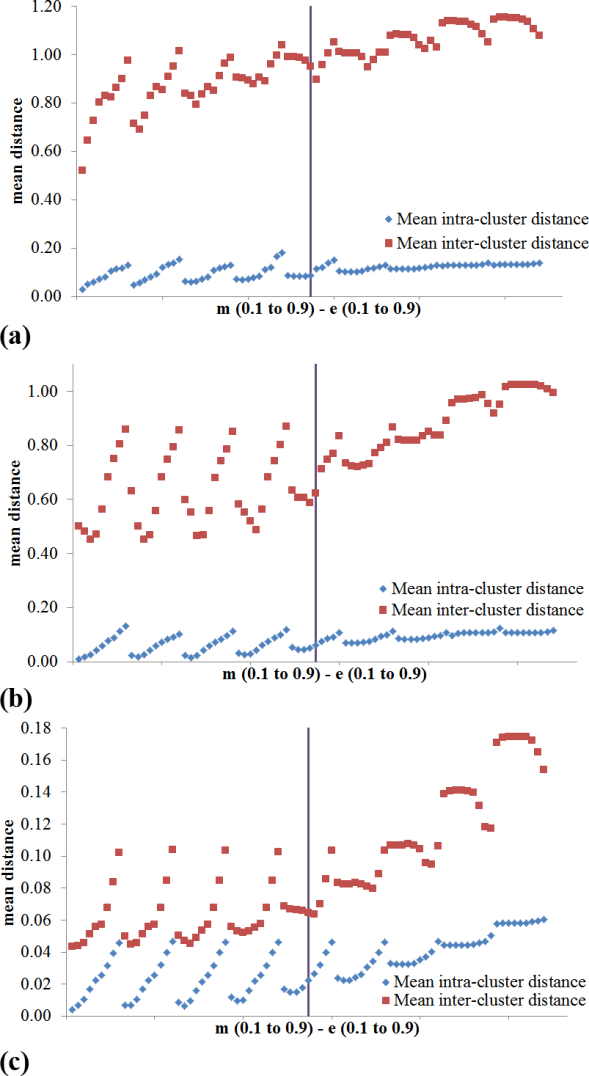


**Figure S5.** Intra- vs Inter-cluster distances over the whole parameter range of merge and extension thresholds. The mean inter- (red squares) and intra- (blue diamonds) cluster distances are provided across a whole range of m and e combinations. The purple perpendicular line indicates the distances at the default parameter settings of m = e = 0.5.


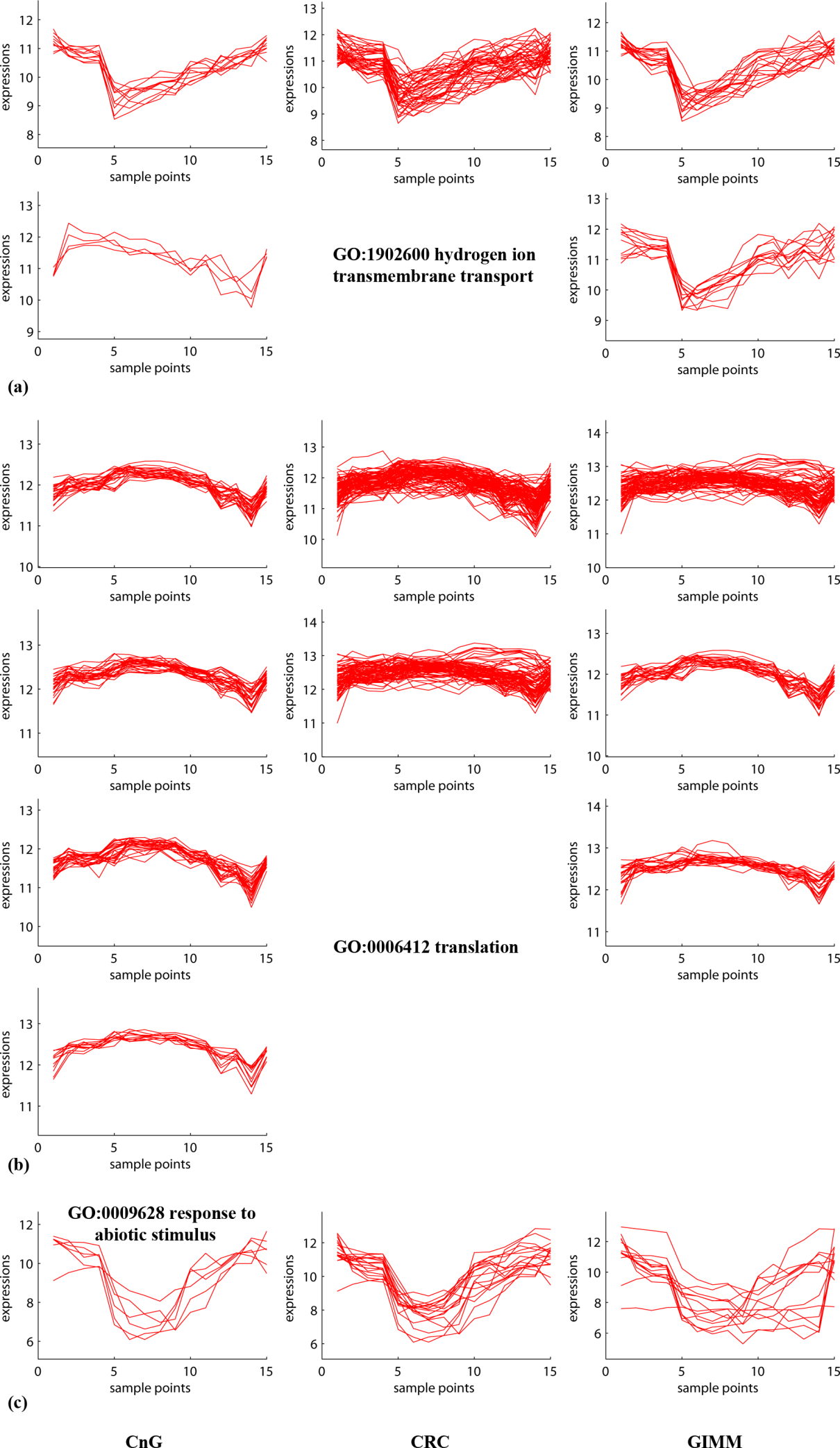


**Figure S6.** Examples of clusters of expression profiles obtained from GLU. Figures along the same column are generated from the results of the clustering analysis carried out by CnG, CRC and GIMM from left to right. Clusters generated by CRC tend to be more populated than those generated by CnG or GIMM, all executed at their default parameters. Clusters enriched with Hydrogen Ion Transmembrane Transport GO Biological Process Term (GO:1902600) are provided in (a). Two different trends (despite being associated with the same GO Term) captured by CnG could not be differentiated by either CRC or GIMM. Clusters enriched with Translation GO Biological Process Term (GO:0006412) are provided in (b). CnG captures trends in expression profiles as well as the differences in expression levels for similar trends in different clusters. A cluster enriched with Abiotic Response to Stress GO Biological Process Term (GO:0009628) captured in CnG is provided with those clusters in CRC and GIMM, which contain the same members along with others, but are not enriched with the GO Term, are provided in (c).


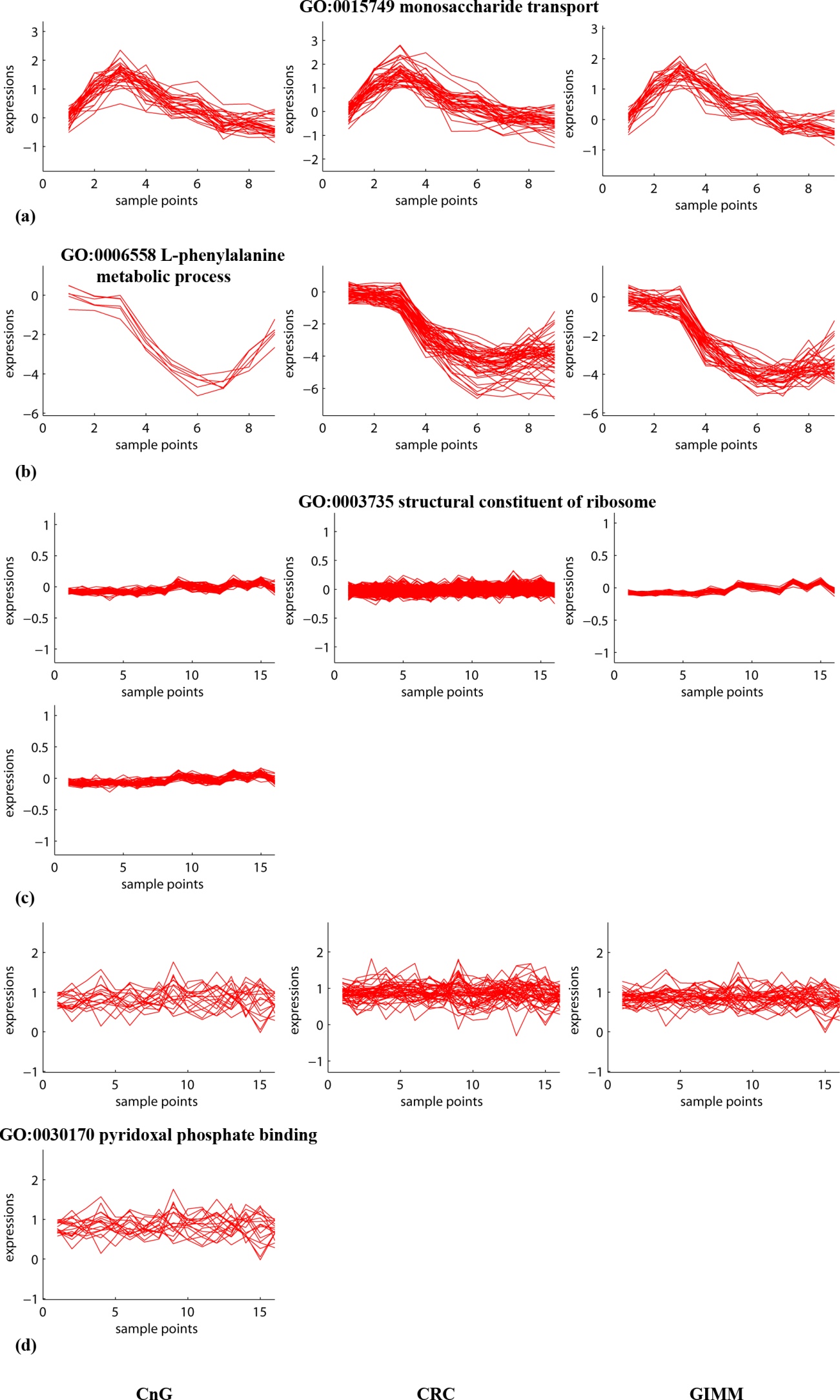


**Figure S7.** Examples of clusters of expression profiles obtained from SPO (a, b) and MUS (c, d). Figures along the same column are generated from the results of the clustering analysis carried out by CnG, CRC and GIMM from left to right. Clusters generated by CRC tend to be more populated than those generated by CnG or GIMM, all executed at their default parameters. Clusters enriched with Monosaccharide Transport GO Biological Process Term (GO:0015749) in SPO are provided in (a). A cluster enriched with L-phenylalanine Metabolic Process GO Biological Process Term (GO:0006558) in SPO captured in CnG is provided with those clusters in CRC and GIMM, which contain the same members along with others, but are not enriched with the GO Term, are provided in (b). Clusters enriched with Structural Constituent of Ribosome GO Molecular Function Term (GO:0003735) in MUS are provided in (c). A cluster enriched with Pyridoxal Phosphate Binding GO Molecular Function Term (GO:0030170) in MUS captured in CnG is provided with those clusters in CRC and GIMM, which contain the same members along with others, but are not enriched with the GO Term, are provided in (d).

**Table S1.** Davies-Boudlin index to determine intra-cluster dispersion and inter-cluster separability

| **Dataset** | **Index** | **CnG** | **CRC** | **GIMM** |
| --- | --- | --- | --- | --- |
| GLU | Optimal/default range/ threshold | 8.39 | 1.45 | 14.02 |
|  | Minimum | 3.83 | 1.45 | 1.40 |
|  | Maximum | 53.52 | 1.45 | 154.77 |
| SPO | Optimal/default range/ threshold | 9.19 | 3.23 | 480.83 |
|  | Minimum | 1.92 | 3.23 | 2.93 |
|  | Maximum | 259.41 | 4.07 | 671.00 |
| MUS | Optimal/default range/ threshold | 21.89 | 20.82 | 260.18 |
|  | Minimum | 5.15 | 20.82 | 25.86 |
|  | Maximum | 1270.17 | 22.45 | 1409.45 |
